# HPRT Deficiency Coordinately Dysregulates Canonical Wnt and Presenilin-1 Signaling: A Neuro-Developmental Regulatory Role for a Housekeeping Gene?

**DOI:** 10.1371/journal.pone.0016572

**Published:** 2011-01-28

**Authors:** Tae Hyuk Kang, Ghiabe-Henri Guibinga, Theodore Friedmann

**Affiliations:** Department of Pediatrics, Center for Neural Circuit and Behavior and San Diego Rady Children's Hospital, University of California San Diego, San Diego School of Medicine, La Jolla, California, United States of America; National Institute on Aging Intramural Research Program, United States of America

## Abstract

We have used microarray-based methods of global gene expression together with quantitative PCR and Western blot analysis to identify dysregulation of genes and aberrant cellular processes in human fibroblasts and in SH-SY5Y neuroblastoma cells made HPRT-deficient by transduction with a retrovirus stably expressing an shRNA targeted against HPRT. Analysis of the microarray expression data by Gene ontology (GO) and Gene Set Enrichment Analysis (GSEA) as well as significant pathway analysis by GeneSpring GX10 and Panther Classification System reveal that HPRT deficiency is accompanied by aberrations in a variety of pathways known to regulate neurogenesis or to be implicated in neurodegenerative disease, including the canonical Wnt/β-catenin and the Alzheimer's disease/presenilin signaling pathways. Dysregulation of the Wnt/β-catenin pathway is confirmed by Western blot demonstration of cytosolic sequestration of β-catenin during in vitro differentiation of the SH-SY5Y cells toward the neuronal phenotype. We also demonstrate that two key transcription factor genes known to be regulated by Wnt signaling and to be vital for the generation and function of dopaminergic neurons; i.e., Lmx1a and Engrailed 1, are down-regulated in the HPRT knockdown SH-SY5Y cells. In addition to the Wnt signaling aberration, we found that expression of presenilin-1 shows severely aberrant expression in HPRT-deficient SH-SY5Y cells, reflected by marked deficiency of the 23 kDa C-terminal fragment of presenilin-1 in knockdown cells. Western blot analysis of primary fibroblast cultures from two LND patients also shows dysregulated presenilin-1 expression, including aberrant proteolytic processing of presenilin-1. These demonstrations of dysregulated Wnt signaling and presenilin-1 expression together with impaired expression of dopaminergic transcription factors reveal broad pleitropic neuro-regulatory defects played by HPRT expression and suggest new directions for investigating mechanisms of aberrant neurogenesis and neuropathology in LND and potential new targets for restoration of effective signaling in this neuro-developmental defect.

## Introduction

Lesch-Nyhan disease (LND) is a generalized monogenic inborn error of metabolism caused by deficiency of the purine reutilization enzyme hypoxanthine-guanine phosphoribosyltransferase (HPRT) activity. The disease is characterized by two major sets of defects; i.e., systemic purine metabolism expressed as hyperuricemia, gouty arthritis and renal calculi [1], and dysfunction of basal ganglia and other neural pathways associated with the hallmark biochemical defect in HPRT deficiency; i.e., markedly reduced neurotransmitter dopamine (DA) in the basal ganglia in both the human and mouse HPRT-deficient brain and resulting dystonia [2]. Evidence has been presented that the basal ganglia DA defect is associated with intrinsic defects in DA neurons [3], [4]. Although the mechanisms of the purine metabolic aberrations are well understood, the mechanisms by which they lead to neural dysfunction are poorly understood.

Many studies have examined the connection between defective purine salvage and the loss of basal ganglia DA in LND and have suggested a number of possible mechanisms by which HPRT deficiency might lead to defective development or function of the DA pathways and DA neurons, including abnormal nigrostriatal axonal arborization or early axonal and neuronal degeneration [5]–[9], secondary metabolic changes that may increase oxidative stress [10], [11] or impaired proteasomal function and protein mis-folding that may generate molecules particularly toxic in DA neurons [12]. The relationship between HPRT deficiency and impaired DA neuron development has been partially clarified by recent studies that have demonstrated that HPRT deficiency leads to broad alterations of DA neuron-related transcription factors and aberrant neurite outgrowth and cellular morphology in mouse MN9D DA neuroblastoma and human NT2 embryonic carcinoma undergoing neuronal differentiation in vitro [13], [14]. Moreover, a more recent report has confirmed similar transcriptional aberrations in HPRT-deficient human neural stem cells [15]. These published studies point to aberrant generation of DA neurons in HPRT deficiency, but a detailed understanding of the aberrant regulation of DA neuron development and function in HPRT deficiency awaits clarification of the complex interplay among multiple transcription and signaling factors that determines generation and differentiation of the DA pathways and midbrain DA neurons.

We have previously published an initial comparative characterization of the transcriptomes of normal and HPRT-deficient mouse striata and normal and LND human fibroblasts [16]. In those studies, we identified a number of genes and gene sets whose expression is dysregulated in HPRT-deficient mouse striatum. To avoid the inevitable interpretational difficulties caused by genetic heterogeneity of individual patient and normal control samples, we have now examined the transcriptional aberrations in the less complex system of wild type (WT) human fibroblasts and human SH-SY5Y neuroblastoma cells in which HPRT expression is efficiently knocked down by transduction with a retrovirus vector expressing a short hairpin RNA targeted to HPRT. We have identified a number of significantly altered signaling pathways in HPRT-deficient cells, and in this report, we concentrate on aberrations related to the Wnt and presenilin (PS) -1 signaling pathways.

Wnt signaling mediates and controls many aspects of vertebrate development approximately 19 different mammalian Wnt glycoproteins regulate many biological processes including embryonic patterning, stem cell self renewal and differentiation, neurogenesis and neural pathway development, tumorigenesis, and others [17]–[21]. Wnt signaling is now recognized to play a key regulatory role in proliferation and differentiation of DA precursors during ventral midbrain (VM) neurogenesis, affecting many processes required for the development of DA neurons from neural stem cells and specification of their properties and functions, including axon guidance and neurogenesis and synapse formation. Recently, evidence has accumulated that dysfunction of these Wnt signaling pathways plays a role in the induction of damage to the DA pathways at the heart of Parkinson's disease, and special attention has been paid to the key role played by Wnt signaling in regulating DA neurogenesis [22], at least partly through the regulation of key transcription factors such as Lmx1a which in turn activate downstream transcription factors to promote differentiation and maturation of the mesencephalic DA neurons. A variety of Wnt molecules such as Wnt1, Wnt3A, Wnt 5A and others have all been shown to promote different aspects of VM morphogenesis such as enhanced DA precursor differentiation, increased survival, neurogenesis and other effects. Signaling by these and other forms of Wnt occurs largely by three separate major pathways; i.e., the canonical pathway mediated by β-catenin and the two non-canonical WNT-PCP and the Wnt-Ca^++^ pathways [23], [24].

A second signaling pathway with important effects on the Wnt signaling pathway is that associated with PS-1. Defects in this pathway not only play a causal role in forms of familial Alzheimer's disease [25], [26] but also interact with the canonical Wnt signaling pathway by stabilizing β-catenin and regulating its transcription [26]. These interactions between the Wnt and PS-1 signaling pathways suggest that they may cooperate in the pathogenesis of some human neurodegenerative and neurodevelopmental diseases, possibly even playing a role in LND. If PS-1 dysregulation indeed is a factor in the development of neuropathology in HPRT deficiency, it seems unlikely that its role is through accumulation of a toxic Aβ_42_, since no histopathological evidence has ever been provided for accumulation of Aβ or other storage materials in the brain of LND patients or in the HPRT knockout mouse.

We now provide evidence that HPRT deficiency produces aberrations in both the canonical Wnt/β-catenin signaling as reflected by elevated levels of cytosolic phosphorylated β-catenin, markedly impaired nuclear transport of β-catenin for faithful gene regulatory function and marked instability of the 23 kDa C-terminal fragment (CTF) of PS-1. These aberrations are associated with the down-regulation of major downstream transcription factor effectors of Wnt regulation that are necessary for effective generation of DA neurons, including Lmx1a and Engrailed 1 (En1). These findings are consistent with a working model of HPRT-induced neuropathology in which the primary underlying purine metabolic aberrations characteristic of HPRT deficiency produce coordinate dysregulation of the key Wnt and PS-1 signaling pathways that in turn collaborate to cause aberrant development of DA pathways and disturbed DA neurogenesis, at least partly by dysregulating key neuronal transcription factors necessary for faithful DA neural development. These findings may provide a new understanding of the neuropathology associated with human HPRT-deficiency and may point to new genetic or metabolic targets for delineating the pathogenic mechanisms and potential therapies for this and possibly other neurodevelopmental or neurodegenerative disorders associated with dysregulated Wnt and PS-1 signaling.

## Results

### HPRT knockdown

The degree of HPRT gene knockdown in both the normal human fibroblasts and the SH-SY5Y cells was approximately 90%, as estimated by quantitative PCR (qPCR) measurements and Western blotting analysis (data not shown). Assays for HPRT enzymatic activity showed a reduction of approximately ∼75% in the knockdown fibroblast cells and SH-SY5Y cells (data not shown). The apparently slightly greater degree of transcriptional knockdown of HPRT compared with HPRT protein and quantitative HPRT enzyme activity may reflect the known high level of HPRT protein stability and is consistent with results of a previous study that reported a similar discrepancy between HPRT transcription and protein level [27]. We consider the 90% knockdown of HPRT gene expression to be sufficient to obtain a useful picture of general transcriptional aberrations in HPRT deficiency since we have previously demonstrated that knockdown of this degree was sufficient to dysregulate DA neuron transcription factor genes and to impair aspects of neurogenesis during in vitro differentiation of NT2 cells toward the neuronal phenotype [14].

### Microarray studies

Of the 48,803 markers that we compared in the triplicate experiments described in MATERIALS AND METHODS, we detected a total of 286 genes whose expression was dysregulated by a factor of >2-fold (Table S1). Of that total, 117 were up-regulated and 169 were down-regulated in HPRT knockdown fibroblasts. The down-regulated genes included the HPRT gene that was down-regulated by 5.0–6.6 fold (Table S1), in general agreement with the qPCR results of normal and knockdown cells (above). The data demonstrated in this result have been deposited in NCBI's Gene Expression Omnibus and are accessible through GEO Series accession number GSE24345 (http://www.ncbi.nlm.nih.gov/geo/query/acc.cgi?acc=GSE24345).

### Gene ontology (GO) and gene set enrichment analysis (GSEA)

A number of the major cellular processes dysregulated by HPRT deficiency and identified by GO and GSEA analysis and further characterized by the publicly available PANTHER analytic software (www.pantherdb.org) are listed in Tables 1 and 2. To correlate gene expression changes with overall biological function, the 286 differentially expressed genes were assigned to established GO classifications by GeneSpring GX10. In this classification, we found significantly aberrant 9 GO terms (p<0.05), particularly those related to cellular surface regions or cellular developmental processes (Table 1). For the results of GSEA analysis, the 286 differentially expressed genes were assigned to the functional gene sets of GSEA by GeneSpring GX10. For this clustering, we used the relaxed false discovery rate (FDR) of <0.4 and p≤0.1. With the relaxed FDR, we identified three GSEA gene sets including Alzheimers_disease_dn (Table 1). By means of GeneSpringGX10 analysis of all 286 dysregulated genes indicated above, we consistently identified the Wnt pathway to be one of the most consistently and significantly aberrant in HPRT-deficient cells (Table 2). After analysis with PANTHER, a number of additional pathways were also identified, including a number of pathways relevant to neuronal function particularly that of the Alzheimer's disease/presenilin signaling pathway, as indicated in Table 2. For the purposes of this present study, we chose to focus on the Wnt and PS-1 pathways for further analysis, as described below.

**Table 1:** Table 1 lists the entities identified by GO and GSEA of the 286 gene significantly aberrantly expressed (>2-fold) in HPRT knockdown human fibroblasts.

GO accession	GO Term	* *p*-value
GO:0005576	extracellular region	7.63E-06
GO:0044421	extracellular region part	2.40E-05
GO:0005578	proteinaceous extracellular matrix	9.34E-05
GO:0031012	extracellular matrix	1.60E-04
GO:0022610	biological adhesion	5.27E-03
GO:0007155	cell adhesion	5.27E-03
GO:0048856	anatomical structure development	5.38E-03
GO:0032502	developmental process	5.38E-03
GO:0007275	multicellular organismal development	1.08E-02

Gene Set	*p*-value [Control] vs [Knockdown]	FDR	ES	NES
Brca_er_neg	0	0.13	-0.27	-1.4
Alzheimers_disease_dn	0.1	0.36	0.2	1.2
Stemcell_hematopoietic_up	0	0.38	0.27	1.44

The GO terms and GSEA gene sets are sorted by p-value and by false discovery rate (FDR), from smallest to largest, respectively. The p-values are marked by asterisks and further described beneath the table.

*Benjamini-Yekutieil corrected *p*-value.

**Table 2:** Table 2 lists the pathways identified by GeneSpring GX and Panther Classification System analysis for the 286 significantly altered (<2-fold) genes in HPRT knockdown fibroblasts.

Predicted pathway by GeneSpring GX10	* *p*-value
Wnt	1.76E-02

Predicted pathway by Panther Classification System	** *p*-value
Alzheimer disease-presenilin pathway	1.90E-06
Integrin signalling pathway	7.81E-05
Wnt signaling pathway	3.44E-04
Axon guidance mediated by Slit/Robo	1.67E-03
Cadherin signaling pathway	7.39E-03
Huntington disease	1.32E-02
Plasminogen activating cascade	1.85E-02
Angiogenesis	2.37E-02
Cytoskeletal regulation by Rho GTPase	2.71E-02
Heterotrimeric G-protein signaling pathway-Gi alpha and Gs alpha mediated pathway	4.34E-02
Inflammation mediated by chemokine and cytokine signaling pathway	4.59E-02

The pathways in table 2 are sorted by p-value (from smallest to largest) indicated by the asterisks and further described beneath the table.

*Fisher's exact test.

**Bonferroni corrected *p*-value.

### Dysregulated components of the canonical Wnt signaling pathway

**Figure 1:**
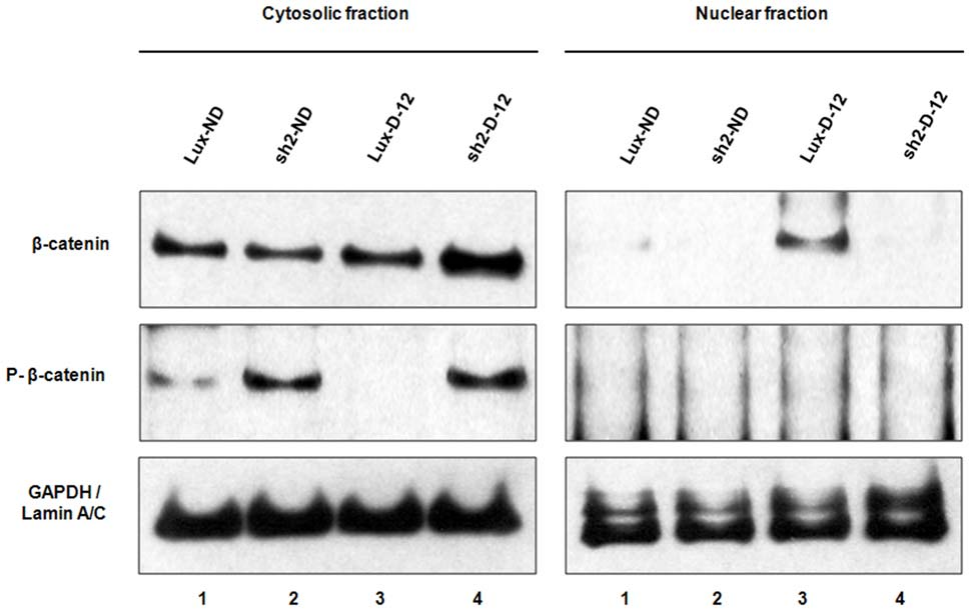
Western blot analysis of total and phosphorylated β-catenin in wild type and HPRT knockdown cells. Undifferentiated control cells transduced with a control luciferase vector (lane 1), undifferentiated cells transduced with the HPRT knockdown vector (2), luciferase-transduced cells at 12 days of differentiation (3) and HPRT-knockdown differentiated cells at 12 days of differentiation (4). Bottom panels represent GAPDH and lamin loading controls.

Because a major mediator of the canonical Wnt signaling is β-catenin, we used Western blotting methods to characterize the content and cellular localization of total and phosphorylated β-catenin in control (Lux-ND) and HPRT knockdown SH-SY5Y cells (sh2-ND) in the basal state and after 12 days of in vitro differentiation, as illustrated in Figure 1. The left panels represent cytosolic β-catenin while those on the right represent the nuclear fractions. In the basal pre-differentiation state, control cells and knockdown cells contain similar levels of cytosolic β-catenin, although the content in knockdown cells is reproducibly slightly reduced. A portion of the β-catenin in both cells is phosphorylated and the amount of phosphorylated protein is markedly increased in the knockdown cells, indicating an apparent increase in phosphorylation of β-catenin in knockdown cells.

After a 12-day period of retinoic acid differentiation, total cytosolic β-catenin in control cells is relatively unchanged, although the amount of the phosphorylated form is moderately reduced. In contrast, HPRT knockdown cells demonstrate a marked increase in both total and phosphorylated cytosolic β-catenin. Although differentiated control cells demonstrate a significant degree of nuclear translocation of unphosphorylated β-catenin, HPRT-knockdown cells reproducibly are markedly deficient in nuclear translocation.

### Effects of aberrant Wnt signaling on downstream transcription factors

**Figure 2:**
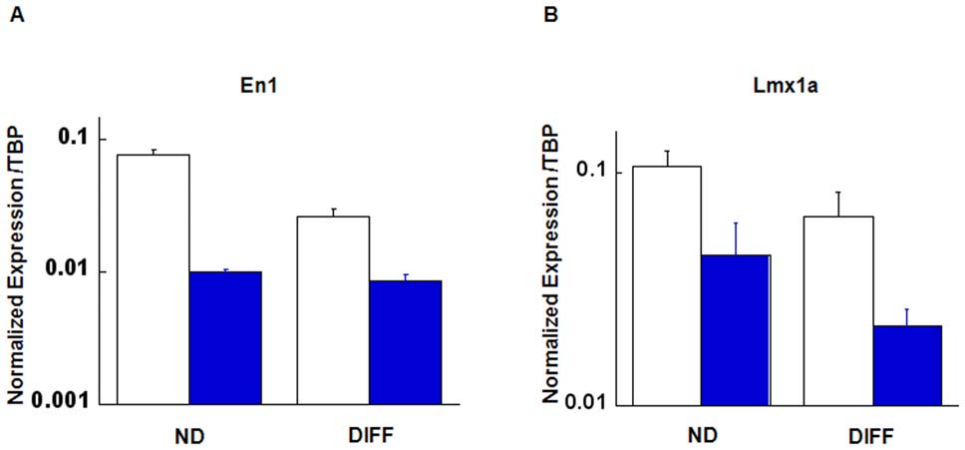
Quantitative PCR analysis of the neural transcription factors En1 and Lmx1a. In both the undifferentiated basal state and after differentiation, there is marked reduction of expression of both transcription factors in the knockdown cells (filled columns). Error bars (Mean + SEM).

The canonical Wnt pathway is one of the key signaling pathways that regulate expression of important transcription factors such as Lmx1a and En1&2 that are necessary for DA neuronogenesis. Because DA deficit is a hallmark defect in HPRT deficiency and because we and other laboratories have previously demonstrated that HPRT deficiency is accompanied by dysregulated expression of several of these important transcription factors [13], [14], including Lmx1a and En1&2, we examined the expression of Lmx1a and En1 in basal and differentiated control and knockdown cells (Figure 2). In both the undifferentiated and the differentiated cells, expression of En1 and Lmx1a is markedly reduced, to levels exceeding 50% in the case of Lmx1a in differentiated cells. This result is consistent with a down-regulated Wnt induction of expression of these transcription factors.

### Aberrant PS-1 expression

The use of the PANTHER analytic method has identified the Alzheimer's disease/presenilin pathway to be the most severely dysregulated pathway in the HPRT knockdown cells (Table 2). Membrane-bound PS-1 is known normally to undergo proteolytic cleavage into N-terminal fragment (NTF) of ca. 30 kDa and CTF of ca. 20 kDa forms that then combine with other components in a gamma-secretase complex to carry out the normal cleavage of amyloid precursor protein (APP) to Aβ. In some forms of familial Alzheimer's disease caused by PS-1 mutations, APP cleavage is aberrant and produces increased levels of the toxic Aβ_42_ cleavage product that accumulates as amyloid deposits characteristic of Alzheimer's disease.

**Figure 3:**
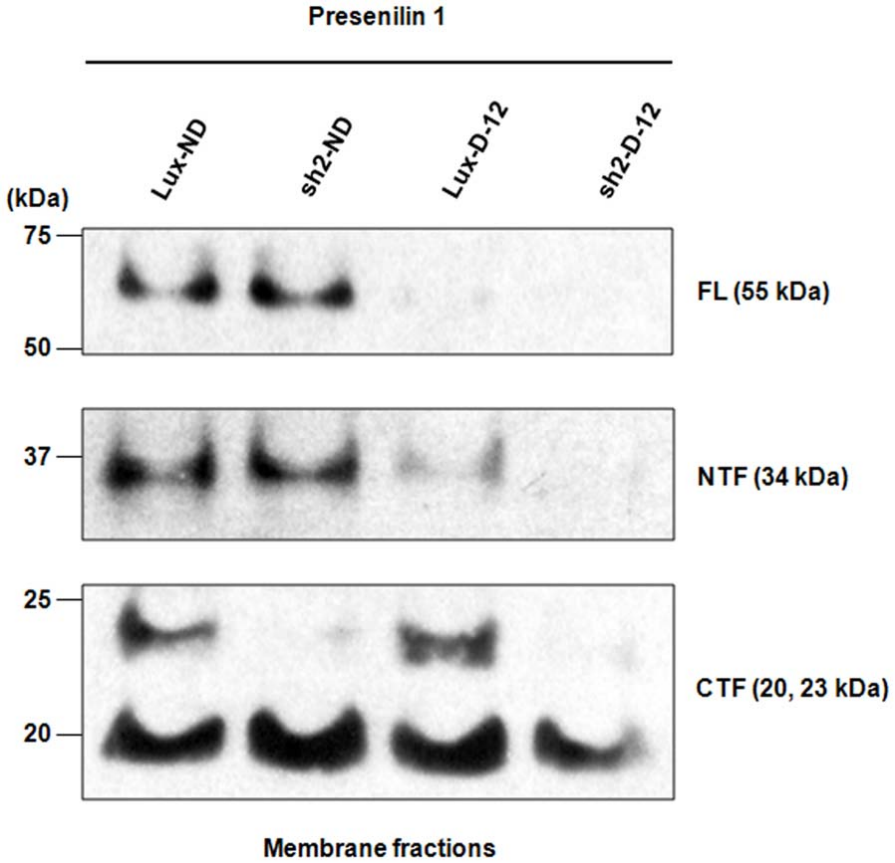
Western blot analysis of PS-1 in wild type and HPRT-knockdown SH-SY5Y cells. Control SH-SY5Y cells were transduced by a retrovirus vector encoding luciferase (Lux-ND) or the anti-HPRT shRNA (sh2-ND) before and after 12 days of differentiation with retinoic acid (Lux-D-12 and sh2-D-12). The 55 kDa full-length PS-1 (FL PS1) and the 34 kDa NTF are present in approximately equal amounts in the undifferentiated control and knockdown cells. After 12 days of differentiation, FL and NTF of PS-1 are markedly reduced. However, at both time, the 23 kDa form of the CTF is markedly reduced in knockdown cells.

To further characterize the mechanism of dysregulated PS-1 expression in HPRT knockdown cells, we used Western blotting methods to identify PS-1 expression in control SH-SY5Y cells before and after differentiation (Figure 3). Both before and after 12 days of differentiation, the amounts of full-length PS-1 and of the NTF protein are similar in the membrane fractions of control and HPRT knockdown cells. In control cells, most of the PS-1 protein exists in the form of the processed ∼30 kDa NTF and 20–25 kDa CTF. However, the HPRT-deficient cells reveal a virtually complete absence of the 23 kDa form of the CTF. Since production of the PS-1 protein and the overall processing seem un-impaired, we interpret the loss of 23 kDa CTF to indicate marked instability of that processed fragment of PS-1 in cells deficient in HPRT expression.

Because the antibody to full-length PS-1 may not recognize the NTF efficiently, we repeated the immunoblotting studies with a second antibody specific for the NTF (see Methods). The results of this study confirmed that the expression of the NTF in undifferentiated HPRT-knockdown cells is indistinguishable from that in control cells but that, in differentiated cells, the amount of NTF is markedly reduced in both cell types, particularly in the HPRT knockdown cells where NTF is virtually undetectable (Figure 3).

**Figure 4:**
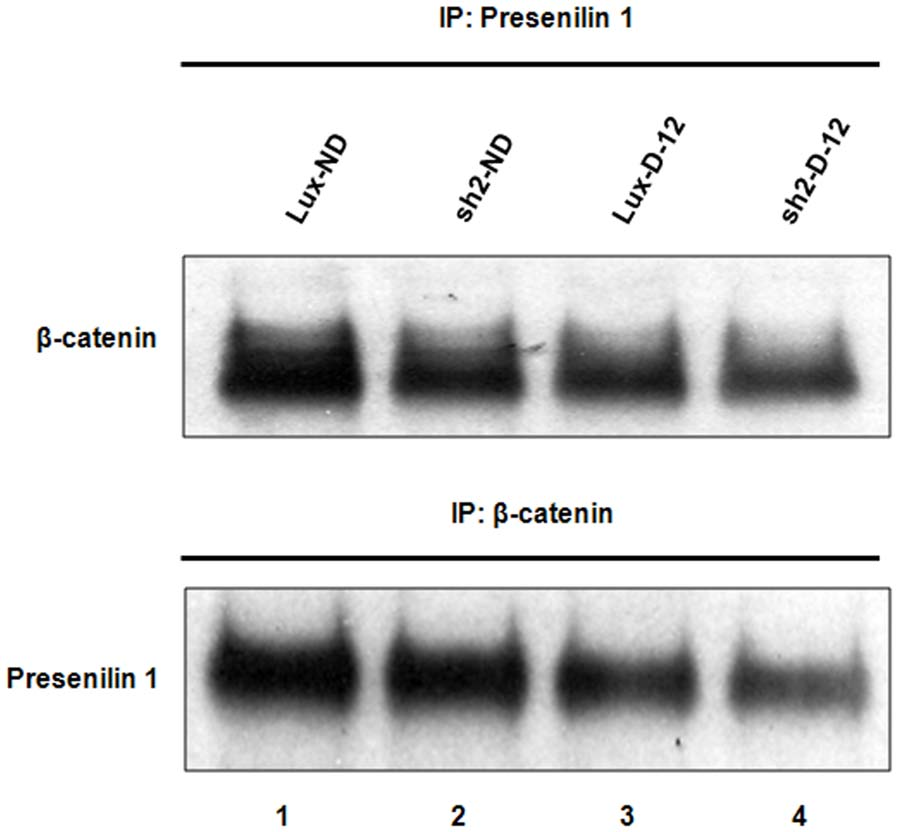
Complexed β-catenin and PS-1 in wild type and HPRT-knockdown SH-SY5Y cells. SH-SY5Y cell proteins were immunoprecipitated by antibody to PS-1 (upper panel) and to β-catenin (lower panel) and reacted with β-catenin or PS-1 antibodies. Un-differentiated control cells transduced by luciferase vector (lane 1), undifferentiated HPRT-knockdown cells (lane 2), 12-day differentiated control cells (lane 3) and 12-day HPRT-knockdown cells (lane 4).

In the present study, we have attempted to characterize a potential physical interaction between Wnt/β-catenin and presenilin-1. It is known that the CTF of PS-1 interacts with and stabilizes β-catenin. A recent study has reported that gamma-secretase activity does not affect PS-mediated regulation of β-catenin [28]. To test the ability of PS-1 and β-catenin to interact and to participate in formation of complexes that might shed light on their coordinate dysregulation by HPRT, we used co-immunoprecipitation methods to determine if HPRT deficiency interferes with the ability of β-catenin and PS-1 function coordinately in a complex. Figure 4 presents Western blots of proteins precipitated either by anti-β-catenin or by anti-PS-1. In both cases, we find that both proteins are immunoprecipitated by either antibody, indicating that they are part of a common complex and may thereby influence their joint functions. The amounts of both proteins are moderately reduced after 12 days differentiation, although the reduction is more pronounced in the HPRT-knockdown cells.

**Figure 5:**
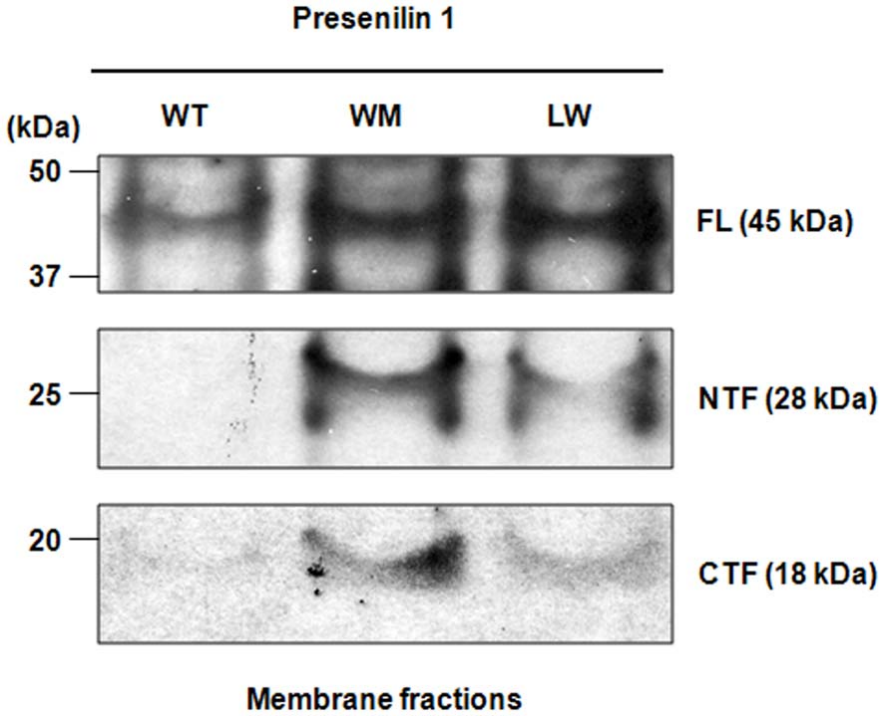
Western blot analysis of PS-1 in fibroblasts from LND patients. WT cells demonstrate very low levels of PS-1 processing to the N- and C-terminal fragment (NTF and CTF, respectively). In both LND cells, processing to both the NTF and CTF proteolytic products is markedly increased compared with that seen in HPRT-positive control WT human fibroblasts.

As further validation of the aberrations of PS-1 signaling in HPRT deficiency, we examined cultures of primary fibroblast from 2 LND patients, one with no detectable residual HPRT activity (WM) and one with 2.5% residual enzyme activity (LW). Figure 5 illustrates the effect of HPRT deficiency on PS-1 expression in these cells compared with control normal human fibroblasts (WT). Most of the PS-1 in WT cells is in the full-length (FL) form and very little PS-1 protein is present either as processed NTF or CTF. In contrast, both LND cells demonstrate markedly increased levels of processed NTF and CTF, indicating a markedly increased level of PS-1 proteolytic processing or proteolytic fragment stability compared with WT cells.

## Discussion

The goals of this study were to identify mechanisms underlying the mechanisms of dysregulated neurogenesis and aberrant DA pathway development in HPRT-deficiency. While our present results do not definitively identify mechanisms of neural disruption in LND, they do identify for the first time several signaling pathways that are markedly dysregulated in HPRT deficiency and that represent important new targets for studying the mechanisms of pathogenesis and possibly even of therapy for this disorder.

Because studies of aberrant gene expression in terminally differentiated cells of different genetic backgrounds often fail to clarify mechanisms that operate in less differentiated developing systems such as those of the developing mammalian CNS, we elected first to identify gene expression aberrations in HPRT-knockdown human fibroblasts, then to validate them in HPRT-deficient human DA cells such as the SH-SY5Y neuroblastoma and then finally to validate candidate gene defects in cells derived from LND patients. Toward these ends, we have used qPCR and Western blotting methods to demonstrate that HPRT deficiency in differentiating human neuroblastoma cells indeed does dysregulate expression of several vital CNS neuronal and developmental signaling pathways in which we had predicted dysregulated expression by GO and GSEA analysis of microarray-based global gene expression analyses of control and HPRT-knockdown human fibroblasts. The extent to which these defects clarify mechanisms of pathogenesis in LND patients is still to be established, but these results demonstrate clearly in fully differentiated normal and LND human fibroblasts and in dopaminergic SH-SY5Y neuroblastoma cells that HPRT plays strong developmental roles in many functions and processes vital for programs key to CNS development, a valuable insight into HPRT neuronal regulatory function whether or not it fully explains the disease phenotype. We conjecture that these robust dysregulations in cultured HPRT-deficient cells may have important neurologically deleterious effects in the human disease.

As we have confirmed by functional studies, the transcriptional characterization of human fibroblasts knocked down for HPRT expression through RNA interference has correctly identified aberrations in HPRT deficiency of a number of important signaling pathways, especially those identified in gene ontology categories as the Wnt signaling and the Alzheimer's disease/presenilin pathways that play roles in the pathogenesis of two common human neurodegenerative and neurodevelopmental disease – Parkinson's and Alzheimer's diseases. Because we found these pathways to be convincingly dysregulated in HPRT-knockdown fibroblasts (Table 2), we extended the gene expression analysis to another cell type; i.e., the SH-SY5Y neuroblastoma cell line that can be differentiated into neuronal cells in vitro. The DA SH-SY5Y neuroblastoma cells have served as valuable and productive model systems to characterize mechanisms of neurogenesis in vitro and, in the present study, convincingly demonstrate major disturbances of expression in HPRT knockdown cells (Figures 1 and 3).

Our current study clearly demonstrates that knockdown of HPRT leads to coordinate dysregulation of several vital signaling pathways that play key roles in regulating aspects of neural pathway development and neurogenesis and for that reason, are attractive candidates for playing an important role in the neurodevelopmental aberrations of LND. For these studies, we have focused on two HPRT-regulated signaling pathways with clear implications for neural function; e.g., the canonical Wnt and the presenilin pathways. Many previous studies have revealed important roles of Wnt signaling in the generation of DA pathways and DA neurons. Of special interest is the known role of the Wnt/β-catenin signaling pathway in regulating hippocampal and midbrain DA neurogenesis [17]–[22]. It is also known that treatment of prenatal DA progenitors with inhibitors of the β-catenin kinase GSK3β enhances DA neurogenesis [29] and that transplantation of rodent fetal neural stem cells treated with Wnt5a results in enhanced survival, differentiation and functional integration of stem cell-derived DA neurons in an animal model of Parkinson's disease [22]. As is clearly shown in Figure 1, HPRT deficiency in SH-SY5Y cells impedes nuclear transport of cytosolic β-catenin where it is required to carry out its gene regulatory roles.

To try to establish a possible direct relevance of these findings in SH-SY5Y cells, we have examined several primary fibroblast cultures from LND patients. In the case of β-catenin, we have so far been unable to identify consistent aberrations in β-catenin expression and nuclear transport, and we are pursuing those studies with larger numbers of control and LND cells. Nevertheless, we infer from these results that the down-regulated Wnt signaling pathway demonstrated in this study may contribute to defective generation and function of DA neurons, possibly by down-regulating downstream transcription factors such as Lmx1a and En-1 (Figure 2) and others whose expression is necessary for effective generation of DA neurons [18]–[21], [30].

In the case of aberrant PS-1 signaling in HPRT-deficient SH-SY5Y cells, we have found little change in the level of full-length or NTF fragment of PS-1 in the HPRT-knockdown cells compared with wild type cells, but virtual absence of the processed 23 kDa CTF in membrane-fractions of HPRT knockdown SH-SY5Y cells, both before and after retinoic acid differentiation (Figure 3). In contrast, LND fibroblasts display little or no PS-1 processing in control WT cells but markedly increased PS-1 processing to the NTF and CTF in the two patients fibroblast cells (Figure 5). These aberrant PS-1 processing steps in HPRT-deficiency LND may provide useful clues to previously unsuspected purinergic contributions to defective processing of APP as found in familial Alzheimer's disease [25], [26], [31], [33]–[35]. We interpret our results to suggest that aberrant PS-1 processing in HPRT deficiency may reveal additional mechanisms of neural damage other than the Aβ_42_ accumulation in Alzheimer's Disease. The NTF and CTF of PS-1 form a heterodimer that is normally incorporated into a gamma-secretase complex whose aberrant function in some forms of familial Alzheimer's disease is implicated in aberrant APP cleavage and over-production of toxic Aβ_42_ or an increased ratio of Aβ_42_ to Aβ_40_ [25]. It is the CTF of PS-1 that has been shown to play a key role in determining pathogenicity in Aβ_42_ accumulation, since it has been shown that Aβ_42_ accumulation does not occur in cells expressing only the mutated NTF [32]. The formation of the PS-1 heterodimer also has been reported to enhance its stability. In the absence of heterodimer formation, it has been thought that the pathogenic CTF is degraded and its pathogenicity reduced or lost. Furthermore, phosphorylation of the CTF of PS-1 [33], in particular the 23 kDa C-terminal isoform has been also reported to decrease Aβ production [34]. Our PS-1 results suggest that a decrease or loss of HPRT expression or mimicking the altered purine levels and pool sizes in HPRT deficiency may destabilize PS-1 heterodimer, allow the degradation of the CTF. We conjecture that purine modulations of purine pathways may eventually provide avenues for therapy or prevention of some neurodegenerative or neurodevelopmental diseases associated with aberrant presenilin-1 function and/or Aβ processing.

As a further test of the functional significance of the PS-1 defect in HPRT-knockdown cells and because PS-1 is known to regulate Notch-1 signaling by cleaving Notch-1, we investigated the effect of HPRT knockdown on the level of Notch intracellular cleavage domain (NICD) in HPRT-deficient SH-SY5Y cells. Our preliminary results indicate that while wild type and HPRT-deficient SH-SY5Y cells have similar levels of NICD in the undifferentiated state, retinoic acid-differentiated HPRT-deficient cells demonstrate a marked (2.4 fold) decrease in NICD (Kang et al., unpublished results). We therefore infer from these results that HPRT may therefore participate in regulating both APP and Notch expression through its effect on PS-1 expression.

In the current case of HPRT effects on signaling pathways, either one of these dysregulated pathways alone; i.e., Wnt signaling and PS-1 expression, may reasonably play a role in at least part of the HPRT deficiency neurological phenotype through dysregulated expression of transcription factors or through mechanisms related to disturbed PS-1 function and gamma-secretase activity. In contrast, the individual roles of β-catenin and PS-1 in neurodegenerative disease together with their coordinate dysregulation by knockdown of the housekeeping gene HPRT suggest that aberrant Wnt and PS-1 pathways may interact and cooperate to produce pathogenic components that individually are subtle but that in the aggregate produce powerfully disrupting effects on vital neural pathways, such as those seen in human HPRT deficiency. It may therefore not be a surprise that the pathways regulating DA neuron development in HPRT deficiency overlap with pathogenic mechanisms found in severe neurodegenerative diseases such as Alzheimer's and Parkinson's diseases. For instance, some defects in the generation and function of DA neurons are obviously part of the Parkinson's disease phenotype but have also been reported in Alzheimer's disease. Loss of dopamine D2 receptors has been reported to play both motor and cognitive roles in Alzheimer's disease [36] and L-dopa administration has been reported to produce a marked reversal of the abnormalities of motor cortical excitability such as decreased intra-cortical inhibition (ICI) in Alzheimer's disease patients [37].

Dysfunctional PS-1 has been reported to negatively regulate the stability of β-catenin for regulating neuronal apoptosis or for preventing neuronal differentiation [38], [39]. This finding is consistent with our finding that both non-differentiated and differentiated HPRT-knockdown cells missing the 23 kDa CTF show lower levels of β-catenin than control cells, possibly because the β-catenin binding site on PS-1 is known to be on the C-terminal portion of PS-1 (residues 322–450) [40]. The interaction between β-catenin and PS-1 may suggest coordinate regulation through formation of multifunctional complexes of these two and possibly other components. Indeed, in our present study, we have confirmed the reported interaction between PS-1 and β-catenin, as demonstrated by co-immunoprecipitation with PS-1 and β-catenin antibodies (Figure 4). The role played by dysfunctional PS-1 in determining the stability and down-regulation of Wnt/β-catenin signaling possibly through impaired complex formation between β-catenin and truncated PS-1 merits further study.

Overall, we interpret our results to indicate that the molecular and neurological phenotypes induced by HPRT deficiency includes wide-spread pleitropic aberrations in components of disparate major signaling pathways, especially dysregulated neurodevelopmental processes associated with Wnt and PS-1 signaling pathways. The possible pathogenic role of individual components of the Wnt/β-catenin and presenilin functions has not previously been demonstrated experimentally to be associated with the development of LND disease. The present study may therefore point toward some common pathways among several quite different neurodevelopmental and neurodegenerative disease and suggest productive new areas of research to understand the neurological defects in both these and other neurodegenerative- and neurodevelopmental-disease.

## Materials and Methods

### Cells and in vitro neuronal differentiation

Human dermal fibroblast-adult (HDF-a) cells were obtained from ScienCell Research Laboratories (ScienCell Research Laboratories, Carlsbad, CA) and were grown to approximately 90% confluence in Dulbecco's modified Eagle's medium (DMEM) high-glucose medium supplemented with 10% (v/v) fetal bovine serum, 1% (v/v) penicillin/streptomycin (Invitrogen, Carlsbad, CA) and 0.2% (v/v) normocin (Invivogen, San Diego, CA) in 5% CO_2_ atmosphere. Medium was renewed every 2–3 days. Human SH-SY5Y cells were obtained from ATTC and were maintained as described above for HDF-a cells. Human SH-SY5Y cells maintained in culture as described above were differentiated with retinoic acid as previously described [41]. Two LND patient fibroblast cells, WM and LW, were provided by Dr. H.A. Jinnah. Department of Human Genetics and Pediatrics, Emory University.

### HPRT-knockdown and control vectors

The small hairpin oligonucleotides for HPRT knockdown were selected as previously described [14]. The selected HPRT hairpin oligonucleotides for this study are as follows: The sequence of the sense element is 5′-ATCCGTGGCCATCTGCTTAGTAGATTCAAGAGATCTACTAAGCAGATGGCCATTTTTTG-3′ and the sequence of anti-sense element is 5′-ATTTCAAAAAATGGCCATCTGCTTAGTAGATCTCTTGAATCTACTAAGCAGATGGCCACG-3′. In both sequences, the hairpin loop in a sequence is underlined. The selected hairpin oligonucleotides were recombined with RNAi-Ready pSIREN Moloney leukemia virus-based retrovirus vectors that express shRNA from the human U6 promoter (Clontech Laboratories, Mountain View, CA), and the pSIREN vectors and plasmid encoding the glycoprotein of vesicular stomatitis virus (pCMV-G) were co-transfected into the packaging cell line GP-293 [14]. The viral titer, production and isolation from packaging cell line GP-293 were carried out in HT-1080 cells as previously described [14]. As a control shRNA, we used an RNAi-Ready pSIREN-RetroQ vector (Clontech Laboratories) expressing a shRNA retrovirus vector targeted against luciferase.

### Cell transduction and selection of HPRT-deficient cells

Control and knockdown cells were infected at a multiplicity of infection (MOI) of approximately 1 with anti-luciferase or anti-HPRT vectors retroviral vectors. Infected cells were grown for 10 days in complete DMEM medium containing 3 ug/ml of puromycin. Bulk cultures were re-plated and maintained in DMEM without puromycin selection for an additional 7 days, after which cells were examined for HPRT expression by transfer to DMEM containing 250 uM 6-thioguanine. The cells infected with the HPRT shRNA retrovirus vector grew and expanded normally in 6-thioguanine, whereas the control cells infected with luciferase shRNA were unable to grow in 6-thioguanine (data not shown).

### RNA purification and quantitative PCR analysis

Total RNAs from HPRT-knockdown and control cells were purified by RNeasy Mini Kit (Qiagen, Hilden, Germany) according to the manufacturer's instruction. To synthesize complementary DNAs from purified RNA, 2 ug of total RNA were applied to the mixture of TaqMan reverse transcription reagents (Applied Biosystems, Foster City, CA) consisting of 5.5 mM MgCl_2_, 500 uM dNTP mixture, 2.5 uM random hexamer, 0.4 U/ml RNase inhibitor and 1.25 U/ml reverse transcriptase in 50 ul of TaqMan RT buffer. Thermal cycling for reverse transcription included activation at 25°C for 10 minutes, reverse transcription at 48°C for 30 minutes and inactivation at 95°C for 5 minutes. For qPCR analysis, the reagents required for the PCR reaction were supplied from a Qiagen QuantiTect SYBR Green PCR Kit (Qiagen) and the PCR reaction was carried out using the Opticon2 System DNA Engine (BioRad, Hercules, CA). Primer sequences for these reactions are summarized in Table S2. For normalization of qPCR, primers specific for the housekeeping gene TATA box binding protein as well as the glyceraldehyde-3-phosphate dehydrogenase were used as a standardization control.

### Immunoblotting

For protein extraction, HPRT-knockdown and control cells were lysed by Mammalian Cell Lysis Kit (Sigma-Aldrich, St. Louis, MO) according to the manufacturer's instructions. For the subcellular fractionation of proteins, HPRT-knockdown and control SH-SY5Y cells and LND patient fibroblast cells were lysed and fractionated by Qproteome Cell Compartment Kit (Qiagen) according to the manufacturer's instructions. Extracted proteins were quantified by the Bradford protein assay. Twenty or Ten microgram protein samples were separated by reducing-Tricine-SDS-PAGE and the separated protein bands were transferred onto polyvinylidene fluoride membrane (Millipore, Billerica, MA) using Mini Trans-Blot Electrophoretic Transfer Cell (BioRad). Blotted polyvinylidene fluoride membranes were blocked by blocking solution containing 1% bovine serum albumin, 20 mM of Tris, 137 mM of NaCl and 0.1% of Tween-20 (Sigma-Aldrich) for one hour at room temperature. Immunodetection of primary antibodies was carried out overnight in a 4°C chamber and signal amplification using horseradish peroxidase-conjugated secondary antibody was performed at room temperature for one hour. As the chemiluminescence reagent, SuperSignal West Pico (Thermo Scientific, Rockford, IL) was treated and the x-ray film was developed by SRX101A film processor (Konica Minolta, Motosu-shi, Gifu-ken). The monoclonal antibody for HPRT (Santa Cruz Biotechnology, Santa Cruz, CA) was used for immunostaining, using goat anti-mouse IgG-HRP (Thermo Scientific) as the secondary antibody. Antibodies for β-catenin, phospho-β-catenin and full length and C-terminal fragment of presenilin-1 were obtained from Cell Signaling Technologies (Danvers, MA) and the antibody for N-terminal fragment of presenilin-1 was purchased from Abcam (Cambridge, MA). Mouse monoclonal antibody to beta-actin (Abcam) and antibodies to GAPDH and Lamin A/C (Cell Signaling Technology) were used as the loading controls. All primary and secondary antibodies were diluted in blocking solution according to manufacturer's instructions. For the co-immunoprecipitation, cells were lysed by 1X cell lysis buffer containing 20 mM Tris (pH 7.5), 150 mM NaCl, 1 mM EDTA, 1 mM EGTA, 1% Triton X-100, 2.5 mM sodium pyrophosphate, 1 mM β-glycerophosphate, 1 mM Na_3_VO_4_ and 1X protease inhibitor cocktail (Roche, Basel, Switzerland). Before immunoprecipitation, the 500 ul of lysate were pre-cleaned by incubation with the 50 ul of protein A agarose beads (50% of bead slurry) at 4°C for 60 minutes. After pre-cleaning, the 500 ul of cell lysate was incubated with anti-β-catenin or anti-presenilin 1 primary antibody at 4°C for overnight. For precipitation of proteins coupled with primary antibody, the 50 ul of protein A agarose beads (50% of bead slurry) was added and incubated with gentle rocking for three hours at 4°C. The precipitate was washed with 500 ul of 1X cell lysis buffer five times and the proteins coupled with protein A agarose beads were eluted by reducing sample buffer for Western-blotting.

### HPRT enzymatic assay

HPRT assays were performed as described [42]. To measure the activity of HPRT, HPRT-knockdown and control cells were treated with lysis buffer (10 mM Tris-HCl, pH 7.4 and 12.5 mM MgCl_2_) and sonicated on ice six times for 10 seconds each interval of 30 seconds. Aliquots of 50 ug of extracted proteins were added to the assay mixture consisting of 2 mM hypoxanthine, 2 mM adenine (Sigma-Aldrich), 0.12 mM [8-^14^C] hypoxanthine, 0.12 mM [8-^14^C] adenine (Moravek Biochemicals, Brea, CA), 0.24 mM AOPCP and 5 mM PRPP (Sigma-Aldrich), and the assay mixtures were further incubated at 37°C for 2 hr. The enzyme reactions were stopped by adding trichloroacetic acid (Sigma-Aldrich) to a final concentration of 2.5% and the supernatants were collected after centrifugation at 10,000 xg for 5 minutes. Aliquots of 3 ul of carrier consisting of 19.1 mM AMP and IMP (1∶1 ratio) from Sigma-Aldrich were spotted onto TLC PEI Cellulose F (EMD Chemicals, Gibbstown, NJ) plates, and 3 ul aliquots of the sample supernatant were applied to the plates. Ascending chromatography was carried with 5% Na_2_HPO_4_ (Sigma-Aldrich) as a solvent and the chromatogram was air-dried. Chromatograms were exposed for 24–48 hours to x-ray film at −70°C. Portions of the plates corresponding to the positions of IMP and AMP were scraped, transferred to scintillation vials and radioactivity was measured using a Beckman LS 6500 liquid scintillating counter (Beckman Coulter, Brea, CA).

### Microarray and data analysis

All reported data are MIAME compliant and that the raw data have been deposited in NCBI's Gene Expression Omnibus (Edgar *et al*., 2002) and are accessible through GEO Series accession number GSE24345 (http://www.ncbi.nlm.nih.gov/geo/query/acc.cgi?acc=GSE24345).a MIAME compliant database (E.g. ArrayExpress, GEO), as detailed on the MGED Society website. For microarray analysis, RNAs from triplicate independent cultures of vector-infected HPRT knockdown and control fibroblasts were prepared separately, pooled and used to prepare cRNA and finally subjected to microarray transcriptional analysis in triplicate. The integrity of total RNA from HPRT knockdown and control cells was confirmed by bioanalyzer (Agilent Technologies, Santa Clara, CA). The quality of total RNA samples from HPRT knockdown and control cells was assessed by 2100 bioanalyzer [43] before application to microarray analysis. We determined that the RNA integrity number (RIN) [44], (maximal degradation  = 1; maximal molecular integrity  = 10) was 10 for both the normal and knockdown cells (data not shown), indicating that isolated RNA samples were of sufficiently high quality to permit subsequent preparation of cDNA for microarray analysis. Microarray transcriptional analysis was performed in triplicate using the HumanWG-6 v3.0 Expression BeadChip system (Illumina, San Diego, CA). All reagents were obtained from HumanWG-6 v.3 Expression BeadChip Kit (Illumina) and all experimental processes were carried out according to manufacturer's instruction (Illumina). After scanning of hybridized BeadChip, quantitation of slide images were performed using Illumina's BeadArray software and the raw data were normalized by Loess normalization method [45], and then the normalized raw data in BeadStudio was exported to GeneSpring GX 10.0.2 (Agilent, Santa Clara, CA). For identification of genes significantly altered in knockdown cell compared with the control normal gene set, total detected entities were filtered by signal intensity value (upper cut-off 100^th^ and lower cut-off 20^th^ percentile) and error (coefficient of variation: CV <50.0 percent) to remove very low signal entities and to select reproducible signal values of entities among the replicated experiments, respectively. In statistical analysis, t-test unpaired (p<0.05) was applied and all significant changes above 2-fold were selected. Signals were selected if they were above microarray background (detection p-value <0.05) in either all six experiments or in at least three knockdown or control experiments. Analysis of GO, GSEA and signaling pathway [46]-[48] was carried out using GeneSpring GX 10.0.2 (Agilent) and the PANTHER Classification System (http://www.pantherdb.org/). In the analysis of signaling pathways using GeneSpring GX 10.0.2 (Agilent), a total of 140 cellular pathways were identified. For GSEA analysis, we used the false discovery rate (FDR) of <0.4 and p≤0.1.

## Supporting Information

Table S1 The list of significantly changed 286 entities (>2-fold)
(XLS)

Table S2 Primer sequences used for qPCR
(XLS)
