# Correction: HPRT Deficiency Coordinately Dysregulates Canonical Wnt and Presenilin-1 Signaling: A Neuro-Developmental Regulatory Role for a Housekeeping Gene?

**DOI:** 10.1371/annotation/893f09fd-ab24-4119-93f4-44a03a3342f9

**Published:** 2011-03-21

**Authors:** Tae Hyuk Kang, Ghiabe-Henri Guibinga, Theodore Friedmann

The authors would like to add H. A. Jinnah as a contributing author. The updated citation is: Kang TH, Guibinga G-H, Jinnah HA, Friedmann T (2011) HPRT Deficiency Coordinately Dysregulates Canonical Wnt and Presenilin-1 Signaling: A Neuro-Developmental Regulatory Role for a Housekeeping Gene? PLoS ONE 6(1): e16572. doi:10.1371/journal.pone.0016572 Affiliation of H. A. Jinnah: Departments of Neurology, Human Genetics, &amp; Pediatrics, Emory University, Atlanta, Georgia, United States of America Contributions of H. A. Jinnah: provided materials/reagents, data analysis, and manuscript preparation. 

